# Principle and Applications of Thermoelectric Generators: A Review

**DOI:** 10.3390/s25082484

**Published:** 2025-04-15

**Authors:** Mohamad Ridwan, Manel Gasulla, Ferran Reverter

**Affiliations:** Department of Electronic Engineering, Universitat Politècnica de Catalunya—Barcelona Tech, 08860 Castelldefels, Barcelona, Spain; mohamad.ridwan@upc.edu (M.R.); manel.gasulla@upc.edu (M.G.)

**Keywords:** autonomous sensor, energy harvesting, low-power electronics, thermoelectric generator

## Abstract

For an extensive and sustainable deployment of technological ecosystems such as the Internet of Things, it is a must to leverage the free energy available in the environment to power the autonomous sensors. Among the different alternatives, thermal energy harvesters based on thermoelectric generators (TEGs) are an attractive solution for those scenarios in which a gradient of temperature is present. In such a context, this article reviews the operating principle of TEGs and then the applications proposed in the literature in the last years. These applications are subclassified into five categories: domestic, industrial, natural heat, wearable, and others. In each category, a comprehensive comparison is carried out, including the thermal, mechanical, and electrical information of each case. Finally, an identification of the challenges and opportunities of research in the field of TEGs applied to low-power sensor nodes is exposed.

## 1. Introduction

Smart technologies play a crucial role in sustainable economic growth as they transform houses, offices, factories, and even cities into autonomic, self-controlled systems that can operate without human intervention [1]. The Internet of Things (IoT) and Wireless Sensor Networks (WSNs) are completely involved in this modernization, offering thousands of small and interconnected devices (so-called sensor nodes) that are capable of sensing and communicating wirelessly [2]. These sensor nodes enable real-time decision-making and automation [3] in many fields, such as Industry 4.0 [4], thus improving factors such as productivity, safety, sustainability, and mobility, and, hence, the people’s quality of life.

A significant challenge in ensuring the sustained operation of sensor nodes is the requirement for a reliable power supply [5]. Currently, three main options exist for powering: (1) electrical-grid connection, (2) primary batteries, and (3) energy harvesters (EH). The first option is limited by placement constraints, as it needs physical connections, thereby compromising flexibility and hindering a truly wireless and autonomous solution. The second option faces limitations in terms of finite energy capacity and the need for replacement that increases both maintenance efforts and operational costs [6]. In addition, batteries are not a sustainable solution with the corresponding recycling costs. An EH, however, is a promising alternative to powering the sensor nodes by capturing “free” energy available from the surrounding environment. This enables the development of self-powered sensor nodes that can operate independently, wirelessly, and sustainably, as reported in numerous studies [7,8,9,10,11,12]. For example, the source of energy was mechanical in [7,9,10], optical in [11], radio frequency (RF) in [12], and hybrid (i.e., optical and thermal) in [8]. In those cases, such energy was employed to power a temperature sensor in [8,12], a health-monitoring system in [9], a microbial-disinfection system in [10], and a gas sensor in [11].

**Figure 1:**
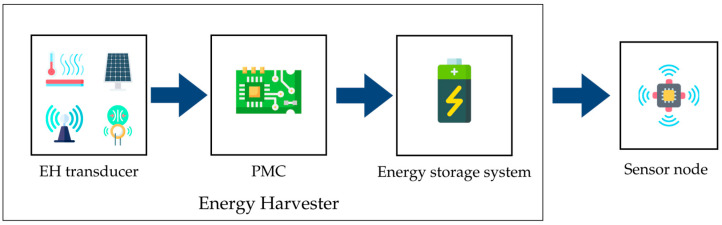
General block diagram of an EH.

An EH, as shown in Figure 1, typically consists of three key blocks: an energy transducer that captures the environmental energy, a power management circuit (PMC) with a power converter, and a control circuit that tracks the maximum power point (MPP) of the transducer [13], and an energy storage system. Apart from being an alternative to primary batteries, this approach also has the potential to complement battery usage and significantly extend their lifespan, enhancing the autonomy and sustainability of sensor nodes [5].

Selecting the appropriate energy source for an EH-based sensor node is essential to achieving an optimal balance between the power generated by the EH and the power consumed by the electronics of the sensor node. It is worth noting that the EH is expected to have a low area (say, around 10–20 cm^2^) in order not to increase the size of the tiny sensor node. In such conditions, the power available at the EH output can be quite low. Fortunately, such a low level of extracted power can be sufficient for low-power sensor nodes, whose average power consumption typically ranges from several microwatts to a few milliwatts [14]. These low levels of power consumption are feasible nowadays thanks to the availability of low-power devices [15,16] and communication protocols [17]. Moreover, the EH has to provide a supply voltage to the sensor node higher than the minimum value required, which is commonly a few units of volt [18].

The same as sensors can acquire information from different energy domains [19], EHs can extract energy from different energy domains, including optical [20,21], thermal [22], mechanical [23], and RF [24]. For optical EH, photovoltaics (PV) is the most established technology for converting optical energy (such as sunlight in outdoor applications or artificial light in indoor applications) into electrical energy. Thermal EHs typically use a thermoelectric generator (TEG) to transform thermal energy (i.e., a gradient of temperature) into electrical energy. Another thermal option is the use of pyroelectric cells [25], but their efficiency is lower than that obtained in TEGs. In the case of mechanical EHs, electrical energy is obtained through electromagnetic [26,27], electrostatic [28], piezoelectric [23,29] or triboelectric [30] transducers. Regarding RF-EH, this captures and converts radio frequency energy into electricity by utilizing rectennas, which are devices that integrate an antenna with a rectifier to efficiently harvest RF signals.

Among the previous harvesting alternatives, optical EHs deliver the highest electrical power density in outdoor scenarios [14], with a power density in the range of 10 mW/cm^2^. However, when deployed indoors, the power density of PV cells decreases significantly (easily, a factor of 100) [31]. Considering such a limitation, a promising alternative for sensor nodes located indoors is the use of thermal EH. According to [14], TEGs offer a wide range of power densities, from 1 µW/cm^2^ to 1 mW/cm^2^. Assuming an exposure area of around 10 cm^2^, the previous power densities result in a power from 10 µW to 10 mW, which is acceptable for many autonomous sensor nodes. However, in order to achieve these values of output power in a TEG, several thermal, mechanical, and electronic issues need to be appropriately handled, such as: (a) the mechanical and thermal coupling of the TEG to the singularities of the thermal source; (b) the proper selection of the dimensions of the heatsink to be attached to the TEG (at the opposite side of the thermal source); (c) the series or parallel configuration (both electrically and thermally) in case that several TEGs are employed; and (d) the tracking of the MPP of the TEG using an appropriate PMC.

Review papers related to TEGs have been recently published in the literature [32,33,34,35,36,37,38]; however, the review in [33] is more related to thermoelectric coolers rather than thermoelectric generators. These review papers, however, show limitations mainly in the following aspects: (a) No electrical model considering the Seebeck, Peltier, and Joule effects was provided, excluding [33] in the context of cooling. (b) The principle of the MPP tracking applied to TEGs was scarcely explained. (c) The thermal management using heatsinks and the configuration of TEGs in an array was not considered, excluding [38]. And (d) the list of applications was not complete. For example, no wearable applications were considered in [32,34,38], no natural heat applications were assumed in [36], and a very reduced number of domestic applications were considered in [37]. Although the list of applications reported in [35] was quite extensive, no information about the heatsink was provided. In addition, the electrical and thermal configuration of the TEGs was not reported in any of the applications in [32,34,35,36,37,38]. Taking this into account, this article aims to bridge this gap by first reviewing in detail the operating principle of TEGs as a generator and then examining applications proposed in the literature in the last years, including information about the heatsink and the electrical and thermal configuration of the TEGs.

The article is structured as follows. Following the introduction in Section 1, Section 2 explains the operating principle of a TEG. Section 3 describes, classifies, and compares applications in which TEGs have been proposed to extract energy, such as domestic and industrial applications. Finally, Section 4 draws the main conclusions and discusses future research directions in the field of thermal EHs.

## 2. Operating Principle of a Thermoelectric Generator

A TEG is a solid-state device that directly converts heat into electrical energy based on thermodynamic principles and effects [39]. The phenomenon of converting heat energy into electrical energy, known as the Seebeck effect, was first discovered in 1821 by Thomas Johann Seebeck [40]. This effect occurs when two dissimilar conductor or semiconductor materials are joined at one end to form a thermocouple, and a temperature gradient is applied between the junction and the open ends. As a result, a voltage difference, the so-called Seebeck voltage, is generated across the open ends of the joined materials due to the diffusion of charge carriers driven by the temperature difference. Thermocouples as a thermal sensing element have been widely employed in the last centuries, especially in environments with very high temperatures. However, thermocouples as an energy harvester (thus resulting in the concept of TEG) have been considered since the mid-20th century [39], thanks to the advancements of the semiconductor technology.

The thermocouples in TEGs are a combination of P-type and N-type semiconductor materials. In a P-type semiconductor, the temperature gradient causes holes to move from the hot side to the cold side, thus creating a charge density difference. On the other hand, in an N-type semiconductor, the process is similar; however, instead of holes, the charge carriers are electrons. These electrons move toward the cold side, causing it to become negatively charged. In steady-state conditions, the charge density difference across the thermocouple is balanced by the temperature difference.

The operating principle of a TEG assuming a single thermocouple is explained in Section 2.1. Afterwards, in Section 2.2, a TEG with several P-N-type pairs electrically connected in series is discussed. Finally, in Section 2.3, ideas for improving TEG performance are provided.

### 2.1. Single-Thermocouple TEG

**Figure 2:**
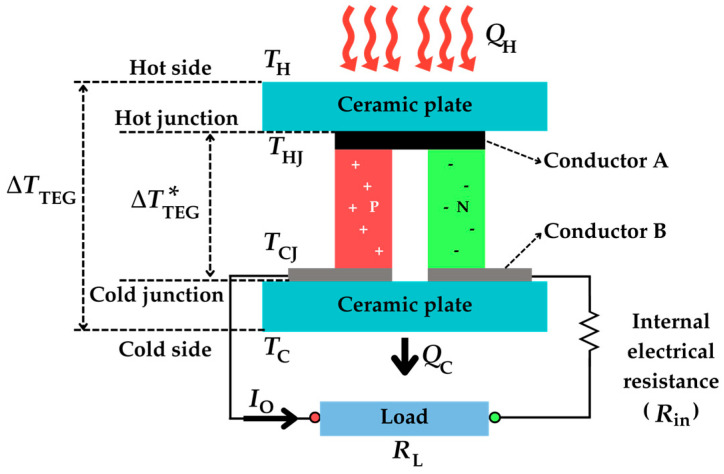
Basic structure of a single-thermocouple TEG.

The fundamental topology of a single-thermocouple TEG is shown in Figure 2. This consists of a single pair of P-N semiconductor materials and two conductors (A and B). The junction at conductor A is known as the hot junction, which is at temperature *T*_HJ_, whereas that at conductor B is the cold junction, whose temperature is *T*_CJ_. The thermocouple is then sandwiched between two ceramic plates, which are electrically insulating and thermally conducting, with a finite thermal conductance *K*. The ceramic plate near the hot junction is considered to be at a temperature *T*_H_, whereas the other one is at *T*_C_.

In open-circuit conditions, an open-circuit voltage (*V*_OC_) appears between the open ends of the thermocouple, resulting from the Seebeck effect, which is proportional to the temperature difference [41], as follows:

(1) $$V_{\mathrm{OC}}=\alpha \left(T_{\mathrm{HJ}}-T_{\mathrm{CJ}}\right)$$

where *α* is the Seebeck coefficient of the thermocouple. This coefficient is equal to *α* = *α*_P_ − *α*_N_, where *α*_P_ and *α*_N_ are the Seebeck coefficients of the P and N-type semiconductors, respectively. For simplicity in the theoretical analysis, it is assumed that the Seebeck coefficient *α*, electrical resistivity *ρ*, and thermal conductivity *k* of the semiconductor materials are temperature-independent. Furthermore, *ρ* and *k* are assumed as the average values of the respective parameters of the P and N-type semiconductors. In addition, it is considered that junctions in the conductors A and B exhibit negligible thermal and electrical resistance. Convection and radiation effects are also neglected in this subsection.

In the topology in Figure 2, thermal energy flows from a heat source to the hot side of the TEG, while the cold side releases the heat to the surroundings. The heat flow rates *Q*_H_ and *Q*_C_, due to thermal conduction, which flow, respectively, from the heat source to the hot junction through the upper ceramic plate and from the cold junction to the surrounding environment through the lower ceramic plate, can be expressed as follows [41]:

(2) $$Q_{H}=\left(T_{H}-T_{\mathrm{HJ}}\right)K$$

(3) $$Q_{C}=\left(T_{\mathrm{CJ}}-T_{C}\right)K$$

In closed-circuit conditions (i.e., when a load resistance *R*_L_ is connected to the thermocouple, as shown in Figure 2), an output current (*I*_O_) is generated that is equal to the following:

(4) $$I_{O}=\frac{V_{\mathrm{OC}}}{R_{\mathrm{in}}{+R}_{L}}=\frac{\alpha \left(T_{\mathrm{HJ}}-T_{\mathrm{CJ}}\right)}{R_{\mathrm{in}}{+R}_{L}}$$

where *R*_in_ represents the internal electrical resistance of the thermocouple, which can be expressed as follows [41]:

(5) $$R_{\mathrm{in}}=\frac{{2\rho h}_{\mathrm{leg}}}{A_{\mathrm{leg}}}$$

where *h*_leg_ and *A*_leg_ denote the height and the cross-sectional area of a thermocouple leg, respectively, and the factor of 2 corresponds to the series connection of the two semiconductors of the thermocouple.

**Figure 3:**
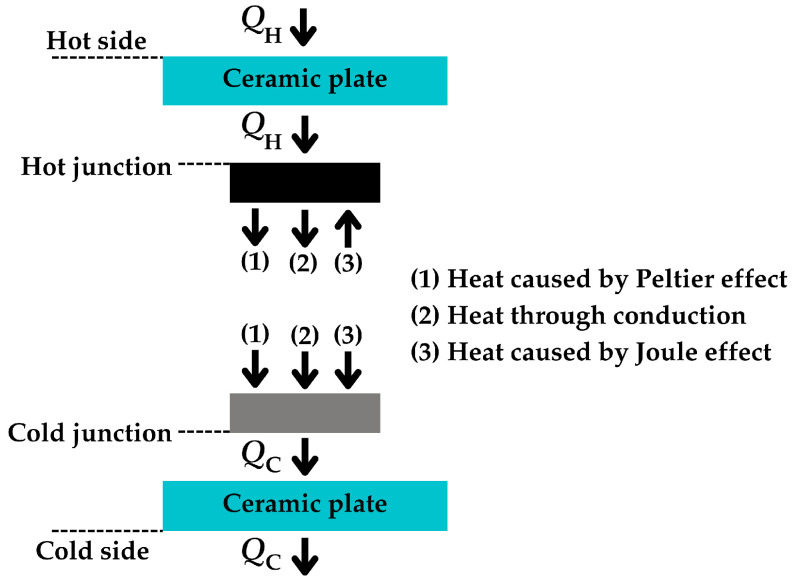
Heat flow rates on hot and cold junctions of a single-thermocouple TEG.

In closed-circuit conditions, the rate of heat exchanged at the hot and cold junctions is the result of the combined effects of three phenomena: thermal conduction, the Peltier effect, and the Joule effect, the last two being caused by the electric current flow. The Peltier effect describes the thermal phenomena occurring at the junction of two different materials when an electric current is applied. Specifically, it is considered the inverse of the Seebeck effect [42], where heat is absorbed or released at the junctions, depending on the direction of the current. On the other hand, the Joule effect is the physical effect by which the passage of current through an electrical conductor produces thermal energy. In terms of heat flow, these effects on the junctions of a single-thermocouple TEG are illustrated in Figure 3. Note that the heat caused by the Peltier effect is released at the hot junction while absorbed at the cold junction [43]. Consequently, there is cooling at the hot junction and heating at the cold junction. Additionally, the heat caused by the Joule effect is absorbed in both junctions, thus causing heating at both junctions. Accordingly, the heat flow rates at both junctions, *Q*_H_ and *Q*_C_, can be expressed as follows [41]:

(6) $$Q_{H}{=\alpha T}_{\mathrm{HJ}}I_{O}+\left(T_{\mathrm{HJ}}-T_{\mathrm{CJ}}\right)K_{\mathrm{in}}-0{.5I}_{O}^{2}R_{\mathrm{in}}$$

(7) $$Q_{C}{=\alpha T}_{\mathrm{CJ}}I_{O}+\left(T_{\mathrm{HJ}}-T_{\mathrm{CJ}}\right)K_{\mathrm{in}}+0{.5I}_{O}^{2}R_{\mathrm{in}}$$

where the first term corresponds to the Peltier effect, the second is related to the thermal conduction between the junctions, and the third to the Joule effect. The positive or negative signs of each term in Equations (6) and (7) show whether the heat is absorbed or released. As for the thermal conduction, this depends on the internal thermal conductance of the thermocouple (*K*_in_) that is obtained as follows [41]:

(8) $$K_{\mathrm{in}}=\frac{{2\mathrm{kA}}_{\mathrm{leg}}}{h_{\mathrm{leg}}}$$

**Figure 4:**
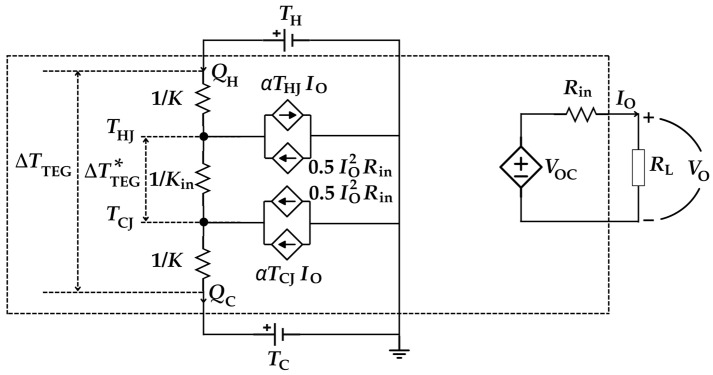
Electrical equivalent model of a single-thermocouple TEG.

Based on the phenomena formulated in Equations (1)–(8), an equivalent electrical model can be developed, as shown in Figure 4 [41,44,45]. Heat flow rate, temperature, and thermal conductance are represented by analogous electrical quantities: current, voltage, and conductance, respectively. The upper voltage source represents the temperature (*T*_H_) at the hot side, while the lower voltage source models the temperature (*T*_C_) at the cold side. The current-dependent current sources describe the contribution of the Joule effect and the Peltier effect to the heat rate at the hot/cold junctions. The right side in Figure 4 represents the electrical output of the thermocouple that is modelled as an equivalent Thevenin circuit, with *V*_OC_ in series with *R*_in_ connected to the load (*R*_L_).

The analysis of the equivalent circuit in Figure 4 provides the effective temperature gradient ($\Delta T_{\mathrm{TEG}}^{\;*}{=T}_{\mathrm{HJ}}-T_{\mathrm{CJ}})$ between the junctions:

(9) $$\Delta T_{\mathrm{TEG}}^{\;*}=\frac{K}{{K+2K}_{\mathrm{in}}+\frac{{2\alpha }^{2}T_{m}}{R_{\mathrm{in}}{+R}_{L}}}\;\Delta T_{\mathrm{TEG}}$$

where Δ*T*_TEG_ = *T*_H_ − *T*_C_ is the external temperature difference across the ceramic plates, and *T*_m_ = (*T*_HJ_ + *T*_CJ_)/2 is the mean temperature of the junctions. It is important to note that $\Delta T_{\mathrm{TEG}}^{\;*}$ depends not only on the thermal and electrical properties of the TEG but also on the load connected at the output. For example, a low-value *R*_L_ leads to an increase in *I*_O_, which, in turn, increases the Peltier effect and reduces$\;\Delta T_{\mathrm{TEG}}^{\;*}$. These effects are described by the third term in the denominator in Equation (9).

The output power (*P*_O_) delivered to *R*_L_ can be expressed as the product of the voltage (*V*_O_) across the load and the current flowing through it, as obtained using the following equation:

(10) $$P_{O}{=V}_{O}I_{O}$$

Considering that *V*_O_ = *I*_O_ *R*_L_, *P*_O_ can also be written as follows:

(11) $$P_{O}{=I}_{O}^{2}R_{L}$$

By substituting Equation (4) in Equation (11), the resulting output power is the following:

(12) $$P_{O}{=V}_{\mathrm{OC}}^{2}\frac{R_{L}}{{\left(R_{\mathrm{in}}{+R}_{L}\right)}^{2}}{=\alpha }^{2}{\left(\Delta T_{\;\mathrm{TEG}}^{\;*}\right)}^{2}\frac{R_{L}}{{\left(R_{\mathrm{in}}{+R}_{L}\right)}^{2}}$$

The same expression could be obtained by calculating *P*_O_ as the difference between *Q*_H_ and *Q*_C_ provided in Equations (6) and (7), respectively.

The electrical performance of a TEG is typically evaluated by means of the output current–voltage (I-V) and power–voltage (P-V) characteristics. The analytical I-V characteristic can be obtained by analyzing the Thevenin circuit shown on the right side in Figure 4, where *I*_O_ can be expressed as follows:

(13) $$I_{O}=\frac{V_{\mathrm{OC}}-V_{O}}{R_{\mathrm{in}}}$$

$\frac{V_{\mathrm{OC}}}{R_{\mathrm{in}}}{=I}_{\mathrm{SC}}$ is the short-circuit current. On the other hand, the P-V characteristic can be found by substituting Equation (13) in Equation (10), resulting in the following:

(14) $$P_{O}=\frac{V_{O}V_{\mathrm{OC}}-V_{O}^{2}}{R_{\mathrm{in}}}$$

**Figure 5:**
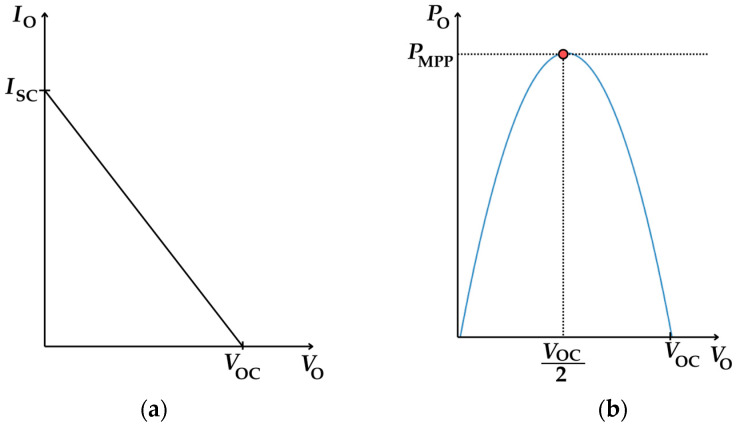
TEG characteristics (**a**) I-V characteristic curve (**b**) P-V characteristic curve.

According to Equations (13) and (14), *I*_O_ has a linear relationship to *V*_O_, while *P*_O_ has a quadratic relationship to *V*_O_, as graphically represented in Figure 5a and Figure 5b, respectively.

In order to deliver the maximum power to the load, the TEG should operate at its MPP. According to the maximum power transfer theorem, the TEG in Figure 4 operates at its MPP when *R*_L_ = *R*_in_, which means that *V*_O_ = *V*_OC_/2, thus generating the following maximum output power:

(15) $$P_{\mathrm{MPP}}=\frac{\alpha^{2}{\left(\Delta T_{\;\mathrm{TEG}}^{\;*}\right)}^{2}}{{4R}_{\mathrm{in}}}$$

This increases with the square of both *α* and $\Delta T_{\mathrm{TEG}}^{\;*}$.

Considering the thermal power (*Q*_H_) applied to the input and the electrical power (*P*_O_) obtained at the output, the TEG efficiency is obtained using the following equation:

(16) $$\eta =\frac{P_{O}}{Q_{H}}$$

According to [38], the maximum efficiency is as follows:

(17) $$\eta_{\max}{=\eta }_{\mathrm{Carnot}}\left(\frac{\sqrt{{1+\mathrm{ZT}}_{\mathrm{avg}}}-1}{\sqrt{{1+\mathrm{ZT}}_{\mathrm{avg}}}+\frac{T_{C}}{T_{H}}}\right)$$

$\eta_{\mathrm{Carnot}}$ is the Carnot efficiency, obtained using the following equation:

(18) $$\eta_{\mathrm{Carnot}}=\frac{T_{H}{-T}_{C}}{T_{H}}$$

where *T*_H_ and *T*_C_ are quantified herein in Kelvin. In Equation (17), *ZT*_avg_ is the (dimensionless) average thermoelectric figure of merit defined as *α*^2^*ρ*^−1^*k*^−1^*T*_avg_, where *T*_avg_ is the average (absolute) temperature of *T*_H_ and *T*_C_. Based on Equation (17), it is essential to have Δ*T*_TEG_ and *ZT* as high as possible to achieve a high efficiency. Thermoelectric materials are recommended to have *ZT* ≥ 1 to make them viable for practical applications [46].

### 2.2. N-Thermocouple TEG

**Figure 6:**
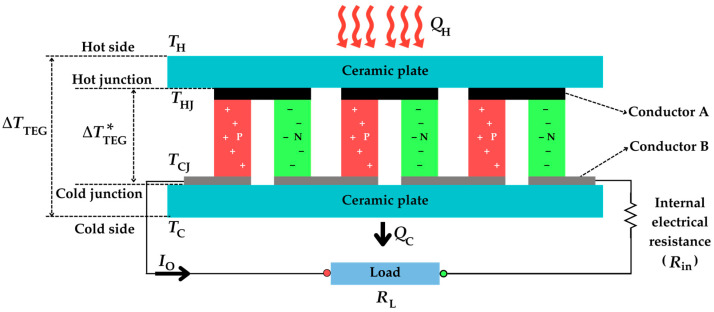
TEG with N thermocouples.

Considering a TEG with *N* thermocouples connected electrically in series and thermally in parallel, as shown in Figure 6, the resulting *R*_in_ and *K*_in_, as given by Equations (5) and (8), respectively, must be multiplied by *N* to reflect the total electrical resistance and thermal conductance of the entire TEG. Additionally, since *N* thermocouples are electrically connected in series, the open-circuit voltage generated by the module becomes *N* times higher, as per Equation (1). The output power, as derived from Equation (12), follows a quadratic relationship with *V*_OC_ and, therefore, it increases by a factor of *N*^2^. Consequently, for a TEG consisting of *N* thermocouples, the relevant equations are adjusted as follows:

(19) $$V_{\mathrm{OC}}=N\alpha \Delta T_{\;\mathrm{TEG}}^{\;*}$$

(20) $$R_{\mathrm{in}}=\frac{{2N\rho h}_{\mathrm{leg}}}{A_{\mathrm{leg}}}$$

(21) $$K_{\mathrm{in}}=\frac{{2\mathrm{NkA}}_{\mathrm{leg}}}{h_{\mathrm{leg}}}$$

(22) $$P_{O}{=N}^{2}\alpha^{2}{\left(\Delta T_{\;\mathrm{TEG}}^{\;*}\right)}^{2}\frac{R_{L}}{{\left(R_{\mathrm{in}}{+R}_{L}\right)}^{2}}$$

(23) $$P_{\mathrm{MPP}}=\frac{N^{2}\alpha^{2}{\left(\Delta T_{\;\mathrm{TEG}}^{\;*}\right)}^{2}}{{4R}_{\mathrm{in}}}$$

According to Equations (20) and (23), it is advisable to have a low value of *h*_leg_ to increase *P*_MPP_. However, according to Equations (9) and (21), decreasing the value of *h*_leg_ generates the opposite effects on *P*_MPP_. Therefore, there is an optimal value for that variable.

### 2.3. Enhancing TEG Performance

**Figure 7:**
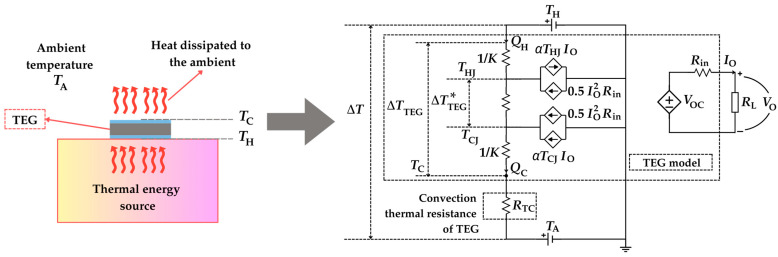
Electrical model of a TEG when heat is dissipated to the ambient through its cold side.

In order to increase the power generation of a TEG, it is important to maximize the value of $\Delta T_{\mathrm{TEG}}^{\;*}$. To do that, it is necessary to maintain a low temperature at the cold side of the TEG. The heat at the cold side of the TEG is dissipated to the ambient mostly through convection. This thermal convection between the cold side and the ambient can be modelled through the law of Newton cooling as follows [47]:

(24) $$Q_{C}=\mathrm{hA}\left(T_{C}-T_{A}\right)$$

where *h* is the convection heat transfer coefficient, *A* is the heat dissipation area, and *T*_A_ is the ambient temperature. For those cases in which *h* is very small, heat exchange with the ambient is also carried out using radiation [48]; however, this scenario is not considered herein. Assuming that *h*, *A*, and *T*_A_ are constant, then *T*_C_ increases so as to dissipate *Q*_C_ until the system reaches an equilibrium condition. Such effects can be modelled via the following convection thermal resistance:

(25) $$R_{\mathrm{TC}}=\frac{\left(T_{C}-T_{A}\right)}{Q_{C}}=\frac{1}{\mathrm{hA}}$$

which is connected in series with the TEG model in Figure 4, as illustrated in Figure 7. A new gradient of temperature is defined herein: Δ*T*, which is the temperature difference between the heat source and the ambient (i.e., Δ*T* = *T*_H_ − *T*_A_). In order to have *T*_C_ as close as possible to *T*_A_ and, hence, increase the output power, *R*_TC_ should be much lower than the other resistances involved in the thermoelectric circuit. To do that, high values of *h* and/or *A* are required, which can be obtained using a heatsink attached to the cold side of the TEG [49], as explained next.

There are five main categories of heatsinks: passive cooling, semi-active cooling, active cooling, liquid cooling, and phase change cooling [49], as summarized in Table 1. In passive cooling, a flat metal made of copper or aluminum is usually employed, while in semi-active cooling, a metallic fin array is utilized. The profile of the fins could be round, triangular, or rectangular, the latter being the most common [49]. The heat dissipation on both passive and semi-active cooling is typically achieved through natural convection. On the other hand, active cooling relies on airflow generated via forced convection. Extra power consumption is needed to power a fan, which is usually installed on a metallic fin array to blow the air. Instead of air, liquid cooling utilizes a liquid to remove the heat. This approach requires additional auxiliary components such as water treatment, valves, pumps, and pipes, and, hence, it is more complicated and expensive. As for phase change cooling, a phase change material (PCM), such as paraffin, is employed to remove the heat since it can absorb a lot of heat before it melts [50]. However, once the PCM reaches its full melting point, the heatsink undergoes a significant temperature rise [51].

**Figure 8:**
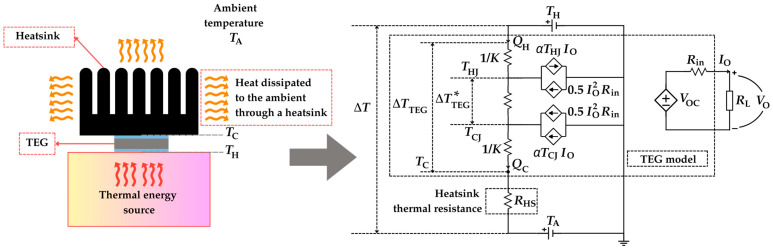
Electrical model of a TEG when heat is dissipated to the ambient through a heatsink.

When a heatsink is attached to the cold side of a TEG, a heatsink thermal resistance (*R*_HS_) replaces *R*_TC_ in the electrical equivalent model in Figure 7, as illustrated in Figure 8 [52]. This *R*_HS_ is typically formulated as follows [49]:

(26) $$R_{\mathrm{HS}}=\frac{1}{\eta_{\mathrm{HS}}h_{\mathrm{HS}}A_{\mathrm{HS}}\;}$$

where *h*_HS_, *A*_HS_, and *η*_HS_ are the convection heat transfer coefficient, the heat dissipation area, and the efficiency of the heatsink, respectively. The value of *h*_HS_ is influenced by fluid properties and motion. For instance, water has better properties than air [48], and forced convection is better than natural convection. A heatsink with a high heat dissipation area also helps to decrease the value of *R*_HS_. However, some applications can offer some constraints in volume, and, hence, the size of the heatsink can be limited. There is an additional thermal resistance (not represented in Figure 8; however, it would be in series with *R*_HS_) related to the contact between the TEG cold side and the heatsink. If thermal paste is employed between them, the value of that thermal resistance becomes lower.

**Figure 9:**
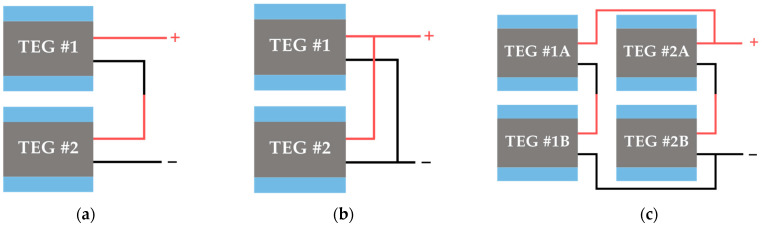
Electrical configuration of TEGs: (**a**) in series, (**b**) parallel, and (**c**) series–parallel.

**Figure 10:**
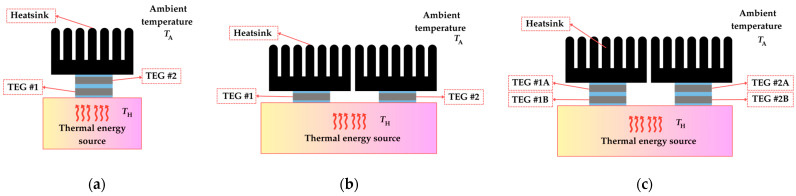
Thermal configuration of TEGs: (**a**) in series, (**b**) parallel, and (**c**) series–parallel.

In case a single TEG is insufficient to provide the power demands of the autonomous sensor, an array of multiple TEGs can be employed. TEG arrays can be arranged in various configurations both thermally and electrically [53], such as series, parallel, or series–parallel configurations. Figure 9 and Figure 10 illustrate the electrical and thermal configurations of TEG arrays, respectively. It is important to highlight that the sum of all individual TEG output power within an array often exceeds the output power of the array itself (i.e., when the TEGs are connected together to form a system). This discrepancy is primarily caused by the nonuniform heat flux distribution at the hot side of the TEG array [54], which means that parameters such as *T*_H_, Δ*T*, *V*_OC_, and *R*_in_ (since this is temperature dependent) are not the same for all the TEGs of the array. The TEGs under less heat flux generate a lower output voltage and current, thus acting as an external load [55]. Under these mismatched conditions, each TEG in the array has a different MPP. Ideally, each TEG should be connected to a dedicated PMC with an MPP tracker (MPPT); however, this approach significantly increases the system's complexity and cost. Conversely, using a single PMC to manage multiple TEGs results in a suboptimal electrical operating point for individual TEGs. Therefore, to address the mismatch problem within the TEG array, it is required to configure the TEG array topology appropriately [54] and determine the optimal number of PMCs relative to the number of TEGs, balancing performance, system complexity, and cost [56].

## 3. TEG Applications

The availability and characteristics of thermal energy sources also influence the feasibility and performance of TEGs. This section reviews the potential of TEGs to harness energy from a wide range of applications, including domestic and industrial environments, as well as specialized areas such as natural heat sources, wearable devices, and other innovative uses. As for the thermoelectric materials, all the references cited in this section that provide material information employ either commercial or custom-designed TEGs based on bismuth telluride (Bi_2_Te_3_).

### 3.1. Domestic Applications

Various thermal energy sources in domestic settings can be utilized, including heat from lamps, household appliances, hot or cold-water piping, window frames, and wood stoves. Table 2 provides a summary of the domestic applications reported in the literature, which are described in more detail in the following paragraphs.

The first subgroup in Table 2 demonstrates the use of TEGs to harvest energy from the heat generated by Light-Emitting Diode (LED) lamps [57,58,59]. In [57], four TEGs connected electrically in series were attached to the surface of a 10-W LED bulb lamp. In the initial experiments without a heatsink, it showed that TEGs could generate a power up to 0.9 mW. However, by optimizing the setup with a heatsink for each TEG, the thermal gradient across the TEGs increased, enhancing the output power to 8.3 mW.

**Figure 11:**
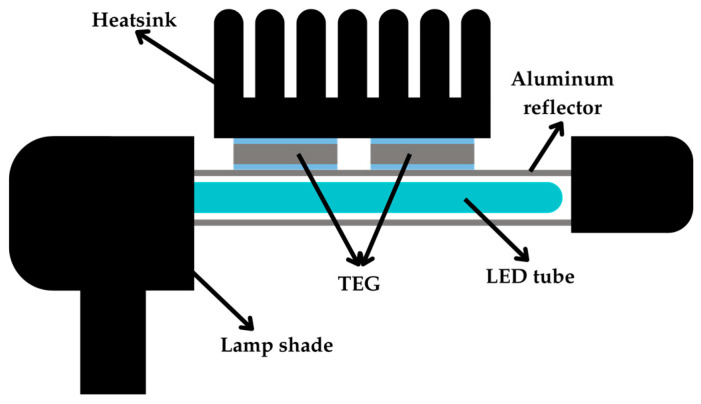
TEG configuration on an LED lamp.

Similar to [57], in [58], two TEGs, connected electrically in series and thermally in parallel with an aluminum heatsink, were attached on an aluminum reflector covering a 13-W LED tube lamp, as shown in Figure 11. It was reported that the output power was 1 mW. According to the author in [58], this low output power was attributed to the low thermal conductance between the LED lamp tubes and the hot side of the TEGs, likely due to the distance between the lamp tubes and the reflector.

In [59], a custom-designed TEG was developed with a smaller size. Four blue LED chips were directly bonded to the hot side of the TEG to improve heat conduction, while a heatsink with a cooling fan was installed on the cold side. The experiments showed that when the LED chips were supplied with a current of 1.4 A, the resulting Δ*T*_TEG_ was 219 °C, which is significantly higher than those reported in previous studies involving LED lamps. Despite this substantial increase in Δ*T*_TEG_, the maximum output power remained comparable, reaching 8.5 mW, with an output voltage of 1.23 V. According to the authors in [59], the output power could be further improved by incorporating highly efficient thermoelectric materials and optimized structures for the chips-on-TEG configuration.

**Figure 12:**
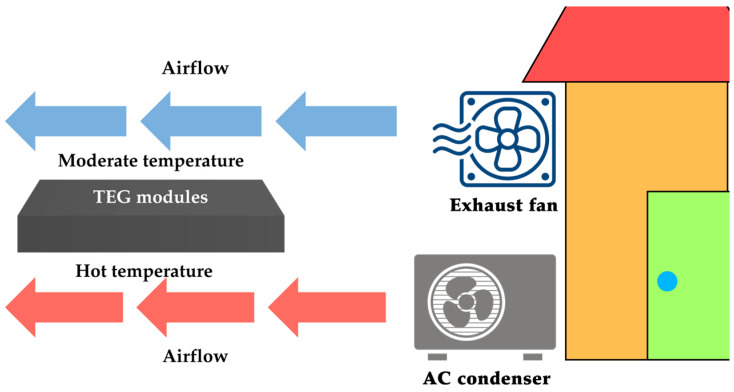
TEG in an AC application using the AC condenser and exhaust fan airflow.

Another subgroup is related to air conditioning (AC) systems [60,61,62,63]. In [60], a simulation-based study was conducted, where several TEGs connected electrically in series were arranged on a 400 × 400 mm^2^ flat plate. The size of each TEG was not reported; however, assuming a typical area of 40 × 40 mm^2^, the number of TEGs on the plate would be 100. In the simulations, each TEG module was heated on the hot side by the hot airflow from an AC condenser (i.e., the external unit of the AC system), while the cold side was cooled by an exhaust fan airflow whose temperature was lower than that from the condenser, as illustrated in Figure 12. The proposed system, thanks to the huge number of TEGs employed, was capable of generating up to 90 W of electrical output power when the space cooling load of the AC was set to 100 kW.

An experimental approach to harvest energy from the hot airflow of an AC condenser was investigated in [61]. This study utilized eight TEGs connected electrically in series, with their hot side attached to an aluminum heatsink that was heated by the hot airflow of the condenser and their cold side attached to three aluminum water-cooled blocks. The results showed that the thermostat setting affected the power generation. When the AC was employed to cool down the indoor temperature, the TEG generated 4.3 mW at a thermostat setting of 20 °C but 3.1 mW at 25 °C.

Other experimental approaches in AC systems were investigated in the compressor [62] and filter drier receiver (FDR) [63]. In [62], two copper plates were clamped to the hot and cold pipes of the compressor unit, with the TEG placed in between, acting as the hot and cold sides, respectively. When the TEG was attached, the hot plate temperature reached approximately 53.9 °C, while the cold plate was at 37.8 °C, thus generating *V*_OC_ = 0.44 V. According to the authors in [62], a higher output voltage could be obtained through an improved installation of the copper plates.

In [63], eighteen TEGs were mounted on an aluminum structure attached to the FDR of an AC unit. The TEGs were arranged in three lines, with six TEGs connected electrically in series in each line to increase the voltage, and the lines were connected electrically in parallel to increase the output current. A heatsink was added to the cold side of the TEGs to enhance heat transfer. The results showed that *V*_OC_ achieved a peak of 5.2 V. However, when connected to the load, the output voltage dropped to 1.26 V (and, hence, the system was not operating at the MPP) and delivered 189 mW.

**Figure 13:**
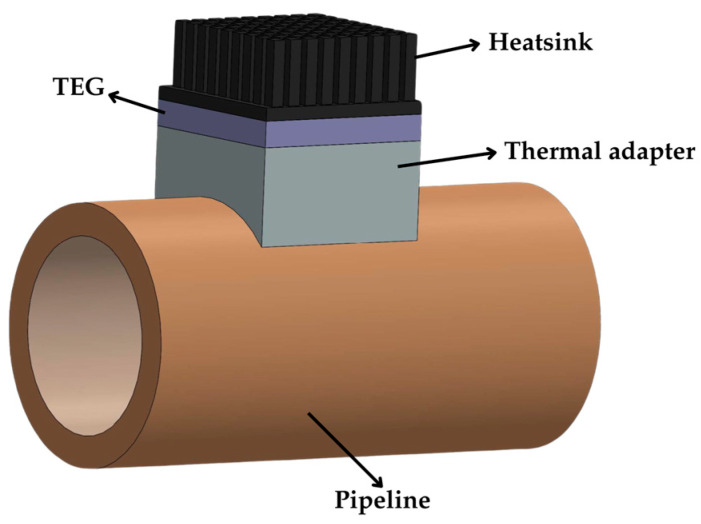
TEG configuration in a pipeline with a thermal adapter.

Water pipelines can also be potential heat sources for TEG applications, as investigated in [64,65,66]. A thermal adapter plate is typically used to address the mechanical mismatch between the TEG and pipeline surfaces, as shown in Figure 13. In [64], both hot and cold water indoor pipelines were studied, creating positive or negative thermal gradients with respect to the ambient. With Δ*T* = ±7 °C, an array of 48 TEGs, connected electrically in series and thermally in parallel with one heatsink for each two TEGs, generated *V*_OC_ = 10 V and *P*_MPP_ = 30 mW under no-wind conditions. Under slow wind conditions (1 m/s), *V*_OC_ and *P*_MPP_ increased by a factor of 2 and 4, respectively, in agreement with Equations (19) and (22).

Another application related to pipelines was studied in [65] to power a water consumption meter. A TEG with a heatsink installed on its cold side was characterized using an electrically controlled heater to emulate the conditions of a pipeline with hot water flowing inside. The investigation was conducted for temperature differences ranging from 1 to 10 °C in relation to the ambient temperature. At Δ*T* = 10 °C, the TEG generated an output power of around 0.35 mW. The system only required a Δ*T* above 4 °C to operate with a single TEG or 2 °C with two TEGs electrically connected in series.

In [66], a non-intrusive ultrasonic water flow system powered by TEGs was suggested, using a water pipeline prototype. Two TEGs, each equipped with a heatsink, were connected to individual voltage boosters linked to a charge controller. Water from a tank was circulated through the pipeline by a pump, with the system harvesting thermal energy from hot water at a Δ*T* between 3 and 12 °C. No output voltages and powers of the TEGs were reported. However, the system was shown to operate with a minimum Δ*T* = 3 °C.

Electric household appliances often generate heat during operation, providing potential thermal sources for TEG applications, as studied in [67,68,69,70]. In [67], a thermal energy harvester with a custom-designed TEG was characterized using a controlled hot plate emulating a wall-mount heater and cooled by a fin heatsink. The hot side temperature ranged from 50 °C to 100 °C, with room temperature at 20 °C. At a typical wall-mount heater surface temperature of 60 °C, the TEG provided *V*_OC_ = 0.47 V and *P*_MPP_ = 4.1 mW.

In [68], a flexible, custom-designed TEG was developed to extract thermal energy from an electric thermos pot. The TEG was placed between two copper plates, and a flexible passive heatsink was used. When the water inside the thermos pot was boiled and maintained at 95 °C, the resulting Δ*T*_TEG_ exceeded 5 °C, thus resulting in *V*_OC_ = 0.11 V and *P*_MPP_ = 0.8 mW.

**Figure 14:**
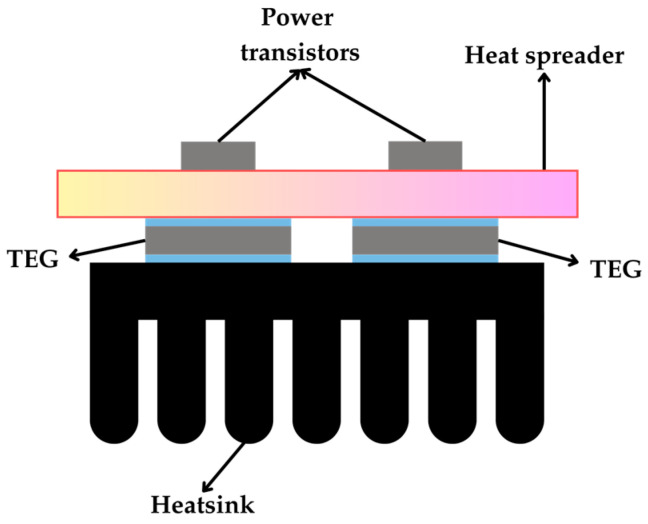
Transistors with a heat spreader and two TEGs connected thermally in parallel.

An alternative household appliance is the power amplifier usually found in audio systems, as discussed in [69]. The heat from the output transistors was leveraged using an aluminum plate as a heat spreader and transferred to the TEGs with a heatsink at the cold side, as illustrated in Figure 14. When the amplifier operated at 50 W, a single TEG provided Δ*T*_TEG_ = 30 °C and *P*_MPP_ = 293 mW. On the other hand, for two TEGs thermally in series, Δ*T*_TEG_ = 50 °C and *P*_MPP_ = 387 mW, and for two TEGs thermally in parallel, Δ*T*_TEG_ = 17.5 °C and *P*_MPP_ = 136 mW. The largest temperature gradient was achieved when the TEGs were connected thermally in series due to the increased thermal resistance. A lower Δ*T*_TEG_ was observed when the TEGs were thermally connected in parallel because of the reduction in the overall thermal resistance of the TEG combination. An improvement of the previous application was carried out in [73].

A microcontroller unit (MCU) controlling any domestic electronic device can also be a potential heat source for TEG applications, as reported in [70]. For the testing, a custom thermal plate platform incorporating two Peltier cells (one to heat the hot side and the other to cool the cold side of the TEG) was utilized, thus ensuring a controlled temperature gradient. The working temperature of the MCU was established by its manufacturer to be between 45 and 81 °C, while the room temperature was between 18 and 32 °C. At the minimum temperature gradient (i.e., 45–32 = 13 °C), the TEG generated *V*_OC_ = 0.47 V and *P*_MPP_ = 12.7 mW.

Window frames [71] and wood stoves [72] have also been considered as thermal energy sources. In [71], a TEG-based energy harvester for WSNs was developed, with four TEGs installed inside a window frame at a 45° angle. Tested under winter conditions, where the outside frame temperature was lower than the inside (room temperature), the TEGs generated a voltage of 0.10 V and a power of 1.5 mW with Δ*T*_TEG_ = 5 °C.

**Figure 15:**
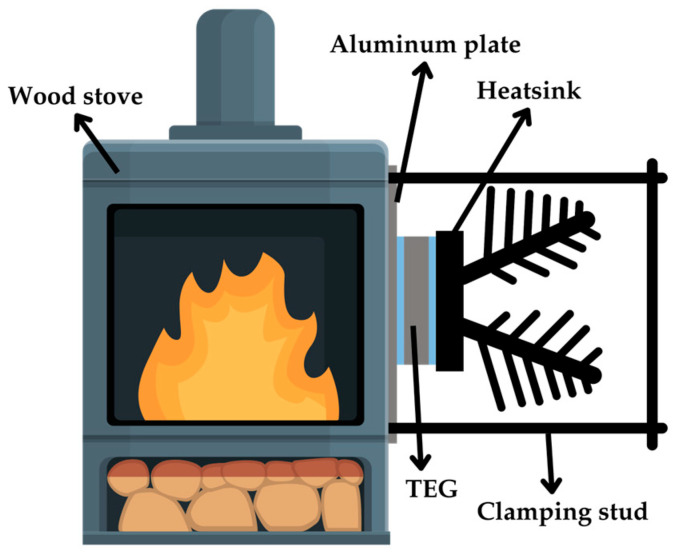
TEG configuration in a wood stove.

In [72], the study involved custom-designed TEGs fitted to the side of a domestic wood stove, with an aluminum plate attached between the TEGs and the stove so as to ensure a good thermal contact, as illustrated in Figure 15. The number of TEG modules mounted under a heatsink was varied from one to three, with the modules electrically connected in series. A single TEG generated *V*_OC_ = 4.1 V and *P*_MPP_ = 4.2 W at Δ*T*_TEG_ = 88 °C. A high output power was obtained, thanks to the high value of Δ*T*_TEG_ and the low thermal resistance of the heatsink employed. When three TEGs were connected thermally in parallel, similar to that in [69], Δ*T*_TEG_ decreased due to the reduction in the total thermal resistance of the TEGs, thus resulting in a reduced output power of 2.7 W per TEG.

### 3.2. Industrial Applications

This subsection reviews thermal energy sources proposed in the literature for industrial environments, including heat from piping systems, high-current conductors, machinery, and industrial wall structures. Table 3 provides a summary of those cases, which are explained in the next paragraphs.

**Figure 16:**
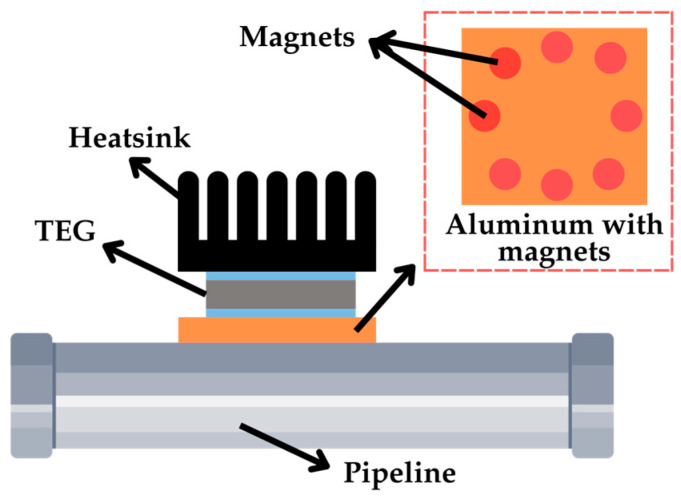
TEG configuration in a pipeline using aluminum with magnets.

The first subgroup is related to piping systems [74,75,76], which involve temperatures that are usually higher (e.g., transporting high-temperature steam) than those found in domestic applications. In [74], a TEG was applied to an industrial pipeline with a contact temperature of approximately 180 °C. Magnets were integrated into an aluminum component to conduct heat from the pipeline to the TEG, as shown in Figure 16. When exposed to a 170 °C heat source, the TEG generated *V*_OC_ ≈ 2.1 V and *P*_MPP_ ≈ 58 mW.

In [75], TEGs for an industrial piping system were studied in simulations and investigated using a heater block and a heat pipe to emulate the industrial piping system. A U-shape adapter coupled two TEGs to the pipe, where the TEGs were connected electrically in series, with each TEG equipped with a heatsink. The experiments showed that with a source temperature of around 248 °C, the system achieved an average Δ*T*_TEG_ from 130 °C to 137 °C, thus producing *V*_OC_ = 8.1 V and *P*_MPP_ = 2.25 W. Such a high value of power was possible thanks to the high Δ*T*_TEG_ and the large heatsink area.

In contrast to previous studies where TEGs were attached to the surface of high-temperature industrial pipelines, in [76], a TEG (intended to power a wireless temperature sensor) was attached to a low-temperature pipeline in a tandem cold mill. In lab experiments, the TEG was tested with a temperature source ranging from 0 to 30 °C. At Δ*T*_TEG_ = 5 °C, the TEG generated *V*_OC_ = 55 mV and *P*_MPP_ = 0.46 mW. However, under real working conditions, the system required a minimum Δ*T*_TEG_ of 7.5 °C to operate properly.

Another subgroup focuses on high-current busbars, which are commonly found in electrical substations [77,78,79]. Due to the ohmic resistance, conductors are heated by the action of the circulating electric current, which generates a temperature difference between the conductor and the environment. In [77], a TEG with a heatsink was attached on a conductor, which was supplied with its rated current of 630 A. Under these conditions, the TEG hot side reached 82.3 °C, and the cold side was at 54.7 °C, thus generating *V*_OC_ = 0.42 V and *P*_MPP_ = 9.2 mW.

Another experimental study explored the power generation of TEGs installed on a tubular busbar in [78]. Due to the large diameter of the busbar, the temperature of the busbar was relatively low. For instance, when supplied with 254 A, the busbar’s temperature remained below 60 °C. Two TEGs, each with a heatsink and connected electrically in series, were mounted on a flat aluminum busbar and heated using heating resistors to emulate real conditions. The TEGs achieved hot and cold side temperatures of 67.7 °C and 57.8 °C, respectively, thus resulting in *V*_OC_ = 0.79 V and *P*_MPP_ = 20.3 mW.

**Figure 17:**
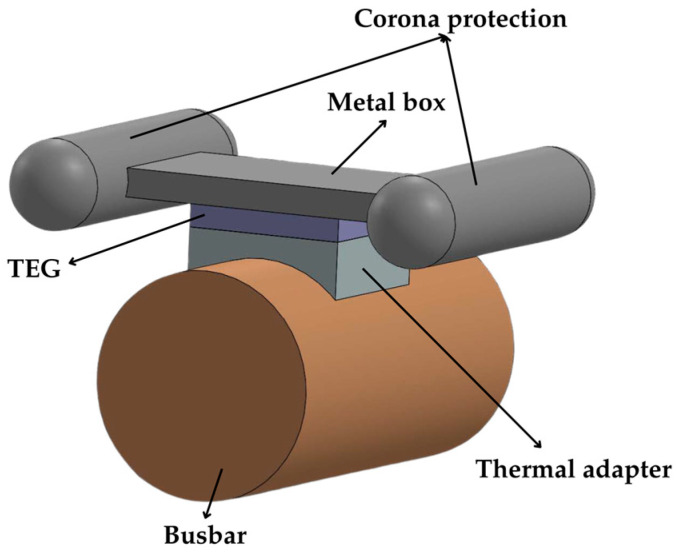
TEG configuration in a busbar with corona protection.

A TEG application on a tubular busbar was also investigated in [79] but with a higher rated current. Two TEGs connected electrically in series and thermally in parallel were employed. Although fin heatsinks could enhance the value of Δ*T*_TEG_, they were unsuitable due to the risk of corona discharge in high-voltage environments. Instead, a metal box enclosure with an aluminum corona protection was used as a heatsink, as shown in Figure 17. With the busbar supplied at 2.85 kA, a temperature difference of 44 °C between the busbar and the ambient was achieved, thus generating *P*_MPP_ = 5.4 mW.

Industrial machinery can also be a potential thermal energy source, as reported in [80]. Four TEGs were installed on the body of a three-phase asynchronous electric motor of a milling machine. A fin heatsink combined with a thin-walled aluminum box filled with paraffin and copper porous foam was attached to the cold side of each TEG. When the motor operated at a constant load of 2 kW, the hot side temperature reached approximately 70 °C, thus resulting in Δ*T*_TEG_ ≈ 19 °C and *V*_OC_ = 0.94 V. When the motor load increased to 2.3 kW with periodic on/off cycles every 360 s, *V*_OC_ reached 1.44 V.

Another industrial application was presented in [81], where two TEGs were installed on a heated plate emulating an industrial hot wall. Each TEG, equipped with a copper extruded fin heatsink, was connected to its own PMC, which were in a cascade configuration to power a WSN node. The plate was heated to 60 °C, a typical temperature in industrial settings. At this condition, the resulting Δ*T*_TEG_ was 14 °C, and TEG #1 generated *V*_OC_ = 0.61 V and *P*_MPP_ = 35 mW, while TEG #2 produced *V*_OC_ = 0.59 V and *P*_MPP_ = 33 mW. Note that a slight variation in *V*_OC_ and *P*_MPP_ might occur even when the same TEG was installed in the same configuration, as heat distribution across the plate might not be entirely uniform.

### 3.3. Natural Heat Applications

Various natural heat sources can be utilized for energy harvesting applications, including heat from the sun, geothermal, and natural spring water. Table 4 provides a summary of these natural heat applications reported in the literature.

The first subgroup is related to solar platforms [82,83,84]. In particular, TEGs can harvest thermal energy using solar-heating panels, as reported in [82,83]. In [82], an anodized aluminum flat panel was installed on a stand and insulated with thin foam to reduce heat losses on its back, on which a TEG was attached. A fin heatsink cooled the TEG, though it still received reflected ground radiation. This setup provided a Δ*T* less than 15 °C, thus resulting in Δ*T*_TEG_ = 7 °C and *V*_OC_ = 0.8 V.

**Figure 18:**
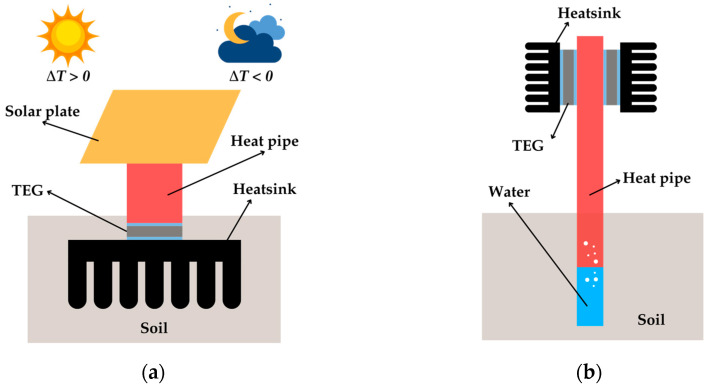
TEG configuration in a (**a**) solar platform and (**b**) geothermal application.

A similar platform with improvements was developed in [83], where the heatsink was buried in the soil instead of being placed in free space, thus avoiding ground-reflected radiation, as illustrated in Figure 18a. A heat pipe of aluminum transferred heat from the solar plate to the TEG with the buried heatsink. Deeper soil depths reduced the heatsink temperature, thus increasing both Δ*T* and Δ*T*_TEG_. At a depth of 40 cm, Δ*T* ≈ 20 °C was reported. Furthermore, this configuration allowed heat transfer to reverse at night, from the soil to the plate, creating a negative Δ*T*.

In [84], a railway track was utilized to extract the heat from the sun instead of using a custom platform. A TEG was attached beneath the railway track with a heatsink partially immersed into the ballasts (rocks), not into the soil. A copper spreader was installed between the railway track and the heatsink and fully exposed to the air. Under these conditions, Δ*T*_TEG_ was 8 °C, thus generating *V*_OC_ ≈ 0.5 V and *P*_MPP_ = 5.8 mW. Meanwhile, in a lab setup, *P*_MPP_ ≈ 317 mW with Δ*T*_TEG_ ≈ 29 °C was reported.

Geothermal energy is another natural heat source that has been considered for TEG applications [85,86,87]. Geothermal heat vaporizes water inside a heat pipe, as shown in Figure 18b. The vapor ascends through the pipe, releasing heat to the TEG placed at the upper part and condensing back via gravity. In [85], geothermal gases at 173 °C enabled six TEGs to achieve Δ*T*_TEG_ = 98 °C, generating *P*_MPP_ = 2.55 W per module and 15.3 W in total. When using 10 TEGs instead, Δ*T*_TEG_ was lower (86 °C) and so was the output power per module (1.88 W) but not the overall power (18.8 W). The study confirmed that adding TEGs could increase the total power but reduce the output power of each TEG module. The high values of output power obtained herein were thanks to the high value of Δ*T*_TEG_, the number of TEG modules, and the large dissipation area provided by the heatsink.

In [86], eight TEGs (mounted on the four sides of a copper sleeve) were utilized to harvest geothermal energy. Unlike in [85], which conducted testing in a subtropical area, the study in [86] carried out experiments in a temperate zone, located between the subtropical and polar regions, with a depth of more than 2 m. During the tests, ambient temperatures ranged from −23 to −10 °C, while the soil temperature remained stable at around 7 °C. The values of Δ*T*_TEG_ ranged from 13 to 25 °C, thus generating a *V*_OC_ from 455 to 722 mV and *P*_MPP_ from 1.6 to 3.6 mW.

Another geothermal case in a temperate zone was studied in [87], where the heat originated from near-surface soil at less than 1 m depth. The setup involved a TEG heated by hot water to emulate soil heat and cooled by air with a heatsink and fan. The experimental results showed that with Δ*T*_TEG_ = 14 °C, the TEG generated *V*_OC_ = 0.7 V. However, in the real case, Δ*T*_TEG_ was typically less than 4 °C, although the temperature difference between the soil and air was 12 °C, resulting in a *V*_OC_ lower than 0.2 V.

Groundwater flowing to the surface as a spring is another natural thermal source, as explored in [88]. The spring water temperature reported in [88] did not freeze even when the ambient temperature decreased below the freezing point of water, maintaining a temperature of approximately 15 °C. Two custom-designed flexible TEGs were used to harvest thermal energy from the spring. The TEGs were attached to a metal heat exchanger immersed in the spring and paired with a flexible heatsink. At an ambient temperature of 30 °C, the TEG generated *P*_MPP_ = 11.4 mW.

Other TEG applications utilizing the temperature difference between water and the ambient were explored in [89,90], considering the fact that water can function as a heat storage system due to its high thermal capacity. In [89], a six-liter water tank inside a thermally insulated box maintained a relatively constant temperature between 22 and 26 °C. Two TEGs were used with two heatsinks on each side, i.e., one immersed in water and the other exposed to air. As the ambient temperature fluctuated between 19 and 31 °C, a temperature difference of 6 °C was obtained, thus generating *P*_MPP_ = 2 mW.

In [90], four TEGs connected electrically in series were used, with one side covered by a 10-mm aluminum plate and the other by an aluminum hollow jacket (jacket #1) filled with water. Two additional jackets were used to store cold water, which replaced the water in jacket #1. The three jackets were appropriately interconnected, and a small pump enabled the circulation of water. It was reported that Δ*T*_TEG_ = 3 °C generated a power of 20 mW.

### 3.4. Wearable Applications

Human body heat is another potential thermal source for TEG applications since its temperature is typically higher than the environment. This heat is commonly harvested using wearable TEGs (WTEGs), which can be comfortably worn on areas such as the forehead, arm, or wrist to power multi-sensor monitoring systems. However, body heat harvesting is constrained by the limited Δ*T* between skin and air (≈5–15 °C depending on ambient temperature). This subsection reviews such wearable applications, with a summary presented in Table 5.

**Figure 19:**
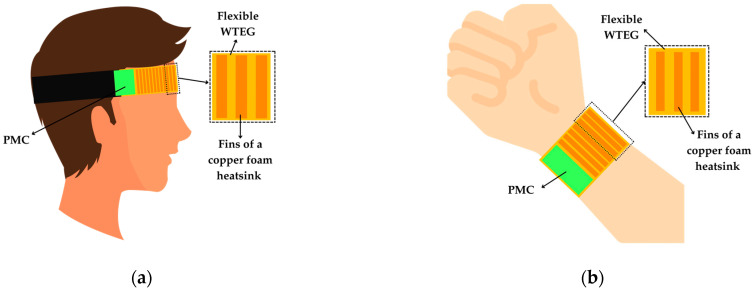
TEG configuration for wearable applications applied to (**a**) forehead and (**b**) wrist.

In [91], a flexible WTEG with a copper–foam heatsink was developed. The WTEG was tested using a body emulator platform set to 37 °C, while ambient temperatures ranged from 18 to 25 °C. Without the heatsink and under no-wind conditions at an ambient temperature of 25 °C, the WTEG generated *V*_OC_ = 14 mV and *P*_MPP_ = 25 μW. When adding the heatsink, *V*_OC_ increased up to 16 mV and *P*_MPP_ to 39 μW. Higher output powers were achieved when it was applied to the forehead, as illustrated in Figure 19a, thanks to the airflow during walking and cycling. It produced 223 mV and 2.8 mW during walking at night (23:00 p.m.) and 390 mV and 8.5 mW during cycling.

A similar approach was demonstrated in [92], where a flexible WTEG with a copper-foam heatsink was developed to harvest thermal energy from the wrist skin, as shown in Figure 19b. When it was tested on a hot plate (from 25 to 65 °C) with an ambient temperature of 20 °C, the WTEG (without the heatsink) provided a *V*_OC_ from 3 to 38 mV and *P*_MPP_ from 1 to 176 μW when Δ*T* varied from 5 to 45 °C. With the heatsink applied, *V*_OC_ was 50 mV, and *P*_MPP_ was 276 μW at Δ*T* = 45 °C. When it was tested on the wrist, the TEG generated *V*_OC_ ≈ 22 mV at Δ*T* = 18 °C, which was achieved by setting the room temperature to 12 °C.

Instead of flexible WTEG, commercial TEGs were utilized for body heat energy harvesting in [93,94]. However, the use of rigid TEGs does not present the portable performance required for better wearing [93]. In [93], three TEGs were stacked to be connected thermally and electrically in series and integrated with a pin-fin aluminum heatsink to harvest thermal energy from wrist skin. At an ambient temperature of 26.3 °C and skin temperature of 33.5 °C, the system produced *V*_OC_ ≈ 110 mV and *P*_MPP_ = 581 μW.

In [94], two types of TEGs, micro-TEG (μTEG) and macro-TEG (mTEG), were tested for body heat harvesting with seven units of each connected electrically in series. The system was evaluated in a temperature-controlled setup. The μTEGs generated a power from 8.5 to 852 μW at Δ*T*_TEG_ between 0.75 and 5.75 °C, whereas mTEGs produced a power from 3.4 to 959 μW at Δ*T*_TEG_ between 1.25 and 5.75 °C. In real-world tests on the wrist, both systems generated an output power of 750–1080 μW while walking outdoors. In a room at 18 °C, they generated 70 μW while sitting and 150–200 μW while walking. In the worst-case scenario, when sitting at 23 °C, both systems could harvest around 50 μW.

In [95], a custom-designed TEG was utilized to harvest thermal energy from the upper arm and chest with copper heat spreaders on both sides. Tested on a hot plate at 37 °C with a room temperature of 18.3 °C, the TEG generated *V*_OC_ = 15 mV and *P*_MPP_ = 5.5 μW, with a power density of 6.1 μW/cm^2^. When applied to the upper arm during walking, the TEG generated 20 μW/cm^2^, which was higher than that obtained at the wrist (13.6 μW/cm^2^) and chest (10.2 μW/cm^2^). Applied on a T-shirt model, the output power ranged from 2 to 8 μW/cm^2^.

**Figure 20:**
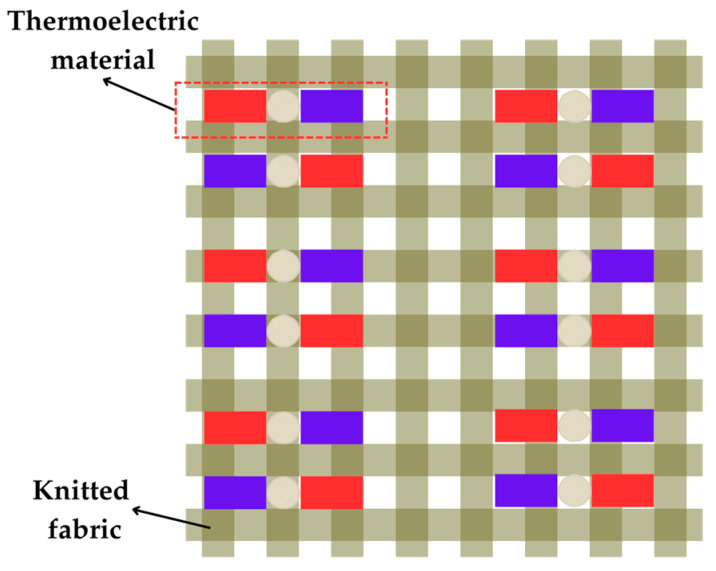
Integration of thermoelectric materials in a knitted fabric.

Textiles used in everyday clothing can also be adapted to fabricate a flexible thermoelectric device architecture, as reported in [96,97]. Garments with thermoelectric elements have been shown to be lightweight, comfortable, and movement-friendly [96]. Thermoelectric materials were knitted into fabrics using yarn, developed in [96], as illustrated in Figure 20. On a heated flat surface, as the temperature increased from 30 °C to 38 °C and Δ*T* rose from 8.8 to 16.4 °C, the resulting *V*_OC_ and *P*_MPP_ increased from 5.4 mV and 0.35 μW to 9.3 mV and 1.2 μW, respectively. When tested on the upper arm, the device produced *V*_OC_ = 7.5 mV and *P*_MPP_ = 0.8 μW.

In [97], thermoelectric materials were integrated into laser-cut cavities of a knitted sportswear fabric. A flexible, 3D-printed heatsink was used to enhance performance and adapt to the fabric’s stretch better than solid heatsinks. Tests conducted indoors at 23 °C evaluated the device on the wrist during stationary and walking conditions. Without the heatsink, *V*_OC_ was approximately 1 mV and 2 mV for stationary and walking scenarios, respectively. With the flexible heatsink, *V*_OC_ increased to 2 mV and 3 mV for the previous two scenarios. The output power was 3.8 μW at *V*_OC_ = 3 mV.

A ring shape TEG was designed in [98] for a wireless earphone to increase its battery backup time using body temperature. The TEG was tested with the earphone’s internal temperature at 32 °C (cold side), and Δ*T*_TEG_ was set to 4.5, 7.5, and 10.5 °C. The TEG produced a *V*_OC_ equal to 23, 37, and 52 mV and an output power of around 110, 310, and 600 μW, respectively. These results were obtained through simulations.

### 3.5. Other Applications

In addition to the previously discussed applications, TEGs have also been applied in other uses, which are summarized in Table 6.

**Figure 21:**
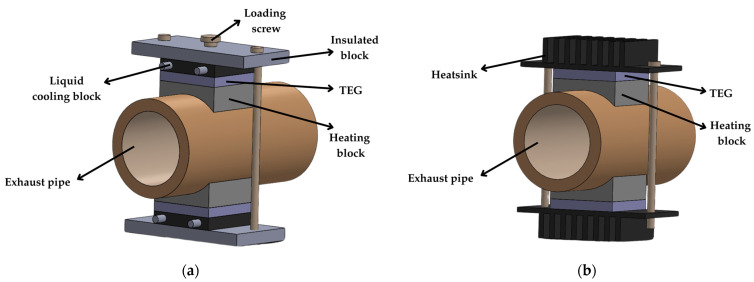
TEG configuration on an automobile exhaust pipe using (**a**) a liquid cooling block and (**b**) a heatsink.

In [99], TEGs were used to harvest thermal energy from automobile exhaust pipes. Twelve pairs of thermoelectric modules were installed, with each pair mounted in a vertical mirror design on the top and bottom surfaces of an exhaust pipe emulator, using liquid cooling, as shown in Figure 21a. Thanks to such a cooling type, these modules generated an average maximum power of 3.78 W per TEG at Δ*T*_TEG_ = 90 °C, with a total power of 90.7 W. In the real automobile exhaust setup, a pair of TEG modules was installed, each with a fin heatsink, as shown in Figure 21b. When the automobile accelerated to 120 km/h, the temperature difference between the heating block and the heatsink was 106.4 °C. At this condition, a single TEG produced 3.12 W and *V*_OC_ = 5.5 V. According to the authors of [99], the reduced output power observed in the real system was attributed to the use of a fin heatsink instead of liquid cooling.

TEGs were also investigated in military applications in [100]. Specifically, TEGs were explored for detecting high-energy laser strikes, which are crucial for the survivability of military assets in future warfare. The proposed TEG-based sensors offered a self-powered passive detection solution for laser weapon systems without interfering with military aircraft’s stealth capabilities. High-energy laser strikes rapidly heated the TEG-irradiated surface, creating a temperature gradient on both sides and a corresponding rise in *V*_OC_. Tests observed TEG behavior under extreme irradiance, showing that irradiated TEGs could generate sufficient power to operate sensor node circuitry. Exposure to an 808-nm, 25-W laser with an 8-mm spot size resulted in Δ*T*_TEG_ = 14 °C and *V*_OC_ = 0.3 V.

Another approach to using TEGs to power an autonomous sensor for fire detection was explored in [101]. The TEG served both as a power supply and as a sensor to estimate Δ*T*_TEG_ based on the TEG output voltage. With an added cold-side temperature sensor, the hot-side temperature was estimated. The TEG was coupled with a fin heatsink, which was installed inside a box containing paraffin. A single TEG generated *V*_OC_ = 1.2 V and *P*_MPP_ = 180 mW at Δ*T*_TEG_ = 40 °C. Using four TEGs connected electrically in series and thermally in parallel, the system produced *V*_OC_ = 2.5 V.

Finally, a TEG was used in [102] to harvest heat from a flameless catalytic burner fueled by lighter fluid, which was able to generate heat through a reaction with a platinum catalyst. At room temperature and in an open space, the hand warmer cap surface of the burner reaches the maximum temperature of 75 °C. Two aluminum fin heatsinks were mounted on either side of the TEG. When tested outdoors in a temperate zone, the burner achieved a maximum temperature at the TEG hot side of around 50 °C, thus resulting in Δ*T*_TEG_ = 11 °C, *V*_OC_ ≈ 0.3 V, and *P*_MPP_ = 12 mW.

## 4. Discussion and Conclusions

According to the review and comparison carried out herein, TEGs show compatibility with various heat sources (including electronic devices, residential and industrial waste heat, natural thermal gradients, and body heat) so that they can be applied across multiple sectors, such as domestic, industrial, and wearable applications. Furthermore, TEGs can be integrated into compact and portable devices, making them particularly useful to power autonomous sensor nodes and IoT devices in environments where conventional power sources may not be feasible. However, as highlighted in this article, the use of TEGs is not exempt from thermal, mechanical, and electrical limitations.

A primary constraint is the strong dependence of TEG performance on maintaining a sufficient temperature gradient and, therefore, an effective thermal management is necessary. Passive or semi-active cooling techniques via a heatsink can be applied to dissipate heat; however, these have a limited heat dissipation capability. Other techniques (such as active, liquid, and phase change cooling) can dissipate heat more efficiently, although these increase the complexity, cost, and/or power consumption of the overall system. The semi-active cooling technique seems the most appropriate for low-power sensor nodes; however, more research oriented toward optimizing TEG–heatsink pairing is required. Although large heatsinks offer a better performance, this has to be well balanced with potential spatial constraints imposed by the application itself. In addition, the type of thermal interface material placed between the TEG and both the thermal source and the heatsink also plays a critical role in the thermal management; however, this has not been evaluated in the literature. These topics are under investigation by the authors of this article.

In order to increase the output power, TEGs can be arranged (both thermally and electrically) in series and/or in parallel; however, this introduces additional trade-offs. When TEGs are configured thermally in parallel, the total thermal resistance decreases, leading to a lower temperature difference across each TEG. Conversely, a thermal series configuration increases the total thermal resistance and maintains a higher temperature difference. However, if there is poor thermal contact between the TEGs connected in series, the contact thermal resistance increases in certain areas, leading to an uneven temperature distribution and, consequently, a reduction in the overall efficiency. From an electrical perspective, a series connection helps to boost the output voltage, while a parallel configuration increases the output current. However, these configurations can generate challenges such as impedance mismatching, which can also degrade power efficiency.

**Figure 22:**
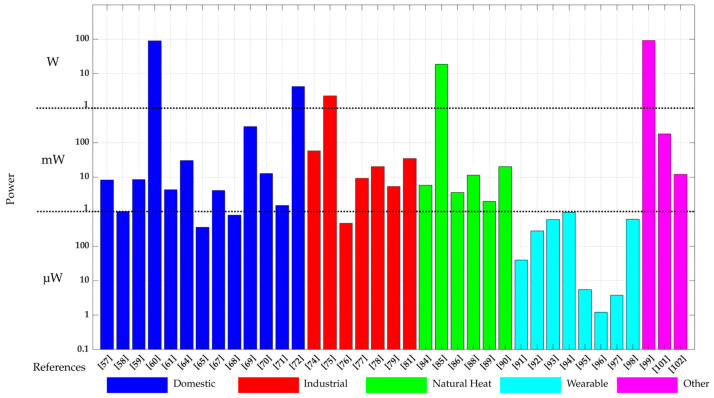
Maximum output power in the TEG applications reported in Section 3 [57,58,59,60,61,64,65,67,68,69,70,71,72,74,75,76,77,78,79,81,84,85,86,88,89,90,91,92,93,94,95,96,97,98,99,101,102].

The design of an efficient PMC capable of handling the inherent low power and voltage levels at the TEG output is also a critical step. As graphically summarized in Figure 22, the output power in most of the applications reviewed in Section 3 ranges from a few units to tens of mWs, which is expected to be sufficient for many autonomous sensor nodes. However, as also inferred from Figure 22, the output power can be in the microwatt range for wearable applications and in the watt range for specific cases with unique testing and operating conditions. The output voltage of a TEG, which usually ranges from tens to hundreds of mV, cannot directly supply the sensor node. Therefore, a PMC is needed to boost the output voltage of the TEG to meet the operational requirements of the electronics of the sensor node. Moreover, another fundamental feature of the PMC is the implementation of the MPPT that continuously adjusts the operating point of the TEG to maximize the power extraction under varying thermal conditions. In that sense, the design of MPPT circuits (for example, based on the fractional open-circuit voltage technique) for TEGs whose power consumption is a small fraction of the harvested power is identified as a future research line. Additionally, when TEGs are being applied to thermal energy sources that can generate both positive and negative thermal gradients (such as pipelines, window frames, and solar platforms), the PMC must be designed to accommodate both positive and negative voltages coming from the TEG. For such cases, no PMC with MPPT capability has been suggested so far in the literature; this is another research line that will be investigated by the authors of this paper in the coming future. Integrating energy storage solutions, such as supercapacitors or rechargeable batteries, is also essential to compensate for intermittent heat availability and maintain continuous power delivery. Addressing these electronic challenges will enable more stable and efficient energy utilization, ensuring that TEGs can be effectively deployed across various real-world scenarios.

With regard to the thermoelectric materials employed, the mainstream in the literature is the use of TEGs based on bismuth telluride. However, the use of such inorganic materials faces important challenges from an environmental point of view. For example, their fabrication involves high temperatures and energy-intensive production methods, leading to high electricity consumption. In addition, tellurium is a scarce element on Earth. For these reasons, the design of TEGs based on greener alternatives and fabrication methods is another future research line. Ideas based on organic polymers have already been suggested, although the *ZT* values provided are lower than those obtained with inorganic materials.

In conclusion, while TEGs offer a substantial potential for waste heat recovery and self-sustaining power generation, their practical implementation still faces various thermal, mechanical, and electrical challenges. Overcoming these challenges requires ongoing research and advancements in TEG configurations, thermal management, and power conditioning techniques. Through further innovation, TEG technology has the potential to become a more viable and scalable solution for sustainable energy harvesting across diverse applications and toward the twin green and digital transition.

## Figures and Tables

**Table 1:** Categories of heatsink cooling methods [49].

Heatsink Type	Thermal Resistance	Complexity	Cost	Additional Requirements	Example
Passive	High	Very low	Very low	NA	Metal plate
Semi-active	Medium	Low	Low	NA	Fin heat sink
Active	Low	Medium	Medium	Additional power for operation	Fan-fin heat sink
Liquid	Very low	Very high	Very high	Additional power for continuous circulation	Liquid cold plate
Phase change	Very low ^(a)^ High ^(b)^	High	High	Additional space	PCM based-heatsink

^(a)^ Before melting point, ^(b)^ after melting point, NA: not applicable.

**Table 2:** Thermal energy harvesting in domestic applications.

Ref.	Thermal Source	Δ*T* (°C)	Δ*T*_TEG_ (°C)	TEG Module	TEG Size (mm)	Heatsink	Heatsink Size (mm)	TEG Config.	*V*_OC_ ^(a)^ (V)	*P*_MPP_ ^(a)^ (mW)
[57]	LED lamp	NR	34	TEC1-12706	40 × 40 × 3.9	Semi active	NR	4 TEGs ^(b)^	0.38	8.3
[58]		NR	2	TGM-199-1.4-1.5	40 × 40 × 4.1	Semi active	102 × 51 × 22	2 TEGs ^(b)^	0.17–0.18	1
[59]		NR	219	Custom	12 × 12 × 3	Active	NR	Single	1.23 V	8.5
[60]	AC	NR	30–134	Custom	NR	NA	NA	NR	NR	19,900–90,300 ^(d)^
[61]		NR	NR	Model 9500	55 × 55 × 3.9	Liquid	NR	8 TEGs ^(b)^	0.47	4.3
[62]		NR	16	1261G-7L31-24CX1	56 × 56 × 5	Liquid	63.5 × 63.5	Single	0.44	NR
[63]		29	NR	SP1848 27154 SA	40 × 40 × 4	Semi active	76 × 105 × 44	18 TEGs ^(c)^	5.2 V	NR
[64]	Pipeline	7	NR	TES1-24102	40 × 40 × 4	Semi active	NR	48 TEGs ^(b)^	10 V	30
[65]		10	NR	TES1-127060	40 × 40 × 3.6	Semi active	NR	Single	NR	0.35
[66]		3–12	NR	GM250-71-14-16	30 × 30 × 3.4	Semi active	40 × 40	2 TEGs	NR	NR
[67]	Wall mount heater	40	NR	Custom	50 × 50 × 3.4	Semi active	NR	Single	0.47	4.1
[68]	Electric thermos pot	NR	>5	Custom, flexible	210 × 75 × 2.5	Passive	NR	Single	0.11	0.8
[69]	Power amplifier	NR	30	GM200-127-14-10	40 × 40 × 3.4	Semi active	65 × 49 × 5	Single	1.7 V	293
[70]	MCU	NR	13	TGM-127-1.0-2.5	30 × 30 × 4.8	NA	NA	Single	0.47	12.7
[71]	Window frame	NR	5	1261G-7L3104CL	30 × 30 × 3.7	Passive	NR	4 TEGs ^(b)^	0.10	1.5
[72]	Wood stove	NR	88	Custom	56 × 56	Semi active	180 × 125 × 136	Single	4.1 V	4200

^(a)^ Corresponding to the TEG configuration. ^(b)^ Connected electrically in series, thermally in parallel. ^(c)^ Connected electrically in a series–parallel configuration, thermally in parallel. ^(d)^ According to simulations. NR: not reported. NA: not applicable.

**Table 3:** Thermal energy harvesting in industrial applications.

Ref.	Thermal Source	Δ*T* (°C)	Δ*T*_TEG_ (°C)	TEG Module	TEG Size (mm)	Heatsink	Heatsink Size (mm)	TEG Config.	*V*_OC_ ^(a)^ (V)	*P*_MPP_ ^(a)^ (mW)
[74]	Pipeline	150	NR	TEG1-30-30-8.5/200	40 × 40 × 3.4	Semi active	51 × 51 × 33	Single	2.1	58
[75]		NR	130–137	Hz-2	29 × 29 × 5	Semi active	125 × 150 × 50	2 TEGs ^(b)^	8.1	2250
[76]		NR	5	CP20151	15 × 15 × 5	Passive	NR	Single	0.055	0.46
[77]	Busbar	NR	28	TEG1-381-1.4-1.2	90 × 30 × 3.41	Semi active	90 × 30 × 25	Single	0.42	9.2
[78]		NR	10	GM250-157-14-16	40 × 40 × 4.1	Semi active	25 × 38 (base), 65 × 40 (top fin field)	2 TEGs ^(b)^	0.79	20.3
[79]		44	NR	TG12-8	40 × 40 × 3.6	Passive	NR	2 TEGs ^(b)^	NR	5.4
[80]	Motor	NR	19	SP1848–271455A	40 × 40 × 4	Phase change and semi active	Phase change box: 40 × 40 × 40, fin heatsink: 40 × 40 × 14	4 TEGs ^(b)^	0.94	NR
[81]	Hot wall	NR	14	TGM287-1.0-1.3	40 × 40 × 3.6	Semi active	88 × 88 × 23	2 TEGs ^(c)^	0.61 (TEG #1) 0.59 (TEG #2)	35 (TEG #1) 33 (TEG #2)

^(a)^ Corresponding to the TEG configuration. ^(b)^ Connected electrically in series, thermally in parallel. ^(c)^ Each one connected to its own PMC. NR: not reported.

**Table 4:** Thermal energy harvesting in natural-heat applications.

Ref.	Thermal Source	Δ*T* (°C)	Δ*T*_TEG_ (°C)	TEG Module	TEG Size (mm)	Heatsink	Heatsink Size (mm)	TEG Config.	*V*_OC_ ^(a)^ (V)	*P*_MPP_ ^(a)^ (mW)
[82]	Solar platform	<15	7	TEG-241.1.0-1.2	40 × 40	Semi active	NR	Single	0.8	NR
[83]		20	NR	TEG-241.1.0-1.2	40 × 40	Semi active	NR	Single	NR	NR
[84]		NR	8	NR	54 × 54 × 3.4	Semi active	225 × 200 × 50	Single	0.5	5.8
[85]	Geo thermal	138–155	86 (A) 98 (B)	TG128LS	40 × 40 × 3.5	Semi active	104 × 28 × 0.3	10 TEGs ^(b)^ (A) 6 TEGs ^(b)^ (B)	NR	18,800 (A) 15,300 (B)
[86]		NR	25	TG122.5	40 × 44 × 3.9	Semi active	NR	8 TEGs ^(b)^	0.7	3.6
[87]		NR	14	TEC1-12706	40 × 40 × 3.2	Active	NR	Single	0.7	NR
[88]	Water	15	NR	Custom, flexible	NR	Semi active	NR	2 TEGs ^(b)^	NR	11.4
[89]		6	NR	CP60440	40 × 40 × 4	NR	NR	2 TEGs ^(b)^	NR	2
[90]		NR	3	TEC1-12706	40 × 40 × 3.2	Liquid	NR	4 TEGs ^(b)^	NR	20

^(a)^ Corresponding to the TEG configuration. ^(b)^ Connected electrically in series, thermally in parallel. NR: not reported.

**Table 5:** Thermal energy harvesting in wearable applications.

Ref.	Thermal Source	Δ*T* (°C)	Δ*T*_TEG_ (°C)	TEG Module	TEG Size (mm)	Heatsink	Heatsink Size (mm)	TEG Config.	*V*_OC_ ^(a)^ (mV)	*P*_MPP_ ^(a)^ (μW)
[91]	Head	12	NR	Custom	35 × 30 × 3	Semi active	NR	Single	16	39
[92]	Wrist	45	NR	Custom	44 × 26 × 2.6	Semi active	NR	Single	50	276
[93]		7	NR	GM200-71-14-16	30 × 30 × 3.4	Semi active	50 × 50 × 20	3 TEGs ^(c)^	110	581
[94]		NR	6	TPG-651 (μTEG) QC32-0.6-1.2 (mTEG)	NR (μTEG) 8 × 8 × 2.6 (mTEG)	Semi active	14 × 14 × 6	7 TEGs ^(b)^	NR	852 (μTEG)959 (mTEG)
[95]	Chest/wrist/arm/t-shirt	19	NR	Custom	13 × 6.6	Passive	20 × 25 × 0.13	Single	15	5.5
[96]	Clothes	16	NR	Custom, knitted	NR	NA	NA	Single	9.3	1.2
[97]		14	NR	Custom	37 × 20	Semi active	NR	Single	3	3.8
[98]	Ear ^(d)^	NR	10	Custom	D = 12 H = 4	NA	NA	Single	52	600

^(a)^ Corresponding to the TEG configuration. ^(b)^ Connected electrically in series and thermally in parallel. ^(c)^ Connected electrically and thermally in series. ^(d)^ Conducted using simulations. NR: not reported. NA: not applicable.

**Table 6:** Thermal energy harvesting in other applications.

Ref.	Thermal Source	Δ*T* (°C)	Δ*T*_TEG_ (°C)	TEG Module	TEG Size (mm)	Heatsink	Heatsink Size (mm)	TEG Config.	*V*_OC_ ^(a)^ (V)	*P*_MPP_ ^(a)^ (mW)
[99]	Vehicle	NR	90	TGM-199-1.4-0.8	40 × 40 × 3.2	Liquid	NR	24 TEGs ^(b)^	NR	90,700
[100]	Laser	NR	14	CP60240	20 × 20 × 4	NA	NA	Single	0.3	NR
[101]	Wildfire	NR	40	TEC12706	40 × 40 × 3.9	Phase change and semi active	Phase change box, fin heatsink: 40 × 40 × 11	Single	1.2 V	180
[102]	Catalytic burner	NR	11	GM250-127-1416	40 × 40 × 4.1	Semi-active	NR	Single	0.3	12

^(a)^ Corresponding to the TEG configuration. ^(b)^ Connected thermally in parallel. NR: not reported. NA: not applicable.

## Data Availability

The data that support the findings of this study are available upon reasonable request from the author.
